# A Convenient and Label-Free Colorimetric Detection for L-Histidine Based on Inhibition of Oxidation of 3,3′,5,5′-Tetramethylbenzidine-H_2_O_2_ System Triggered by Copper Ions

**DOI:** 10.3389/fchem.2021.773519

**Published:** 2021-11-23

**Authors:** Zhikun Zhang, Wenmeng Zhao, Cuixia Hu, Yapeng Cao, Yumin Liu, Qingju Liu

**Affiliations:** ^1^ School of Chemical and Pharmaceutical Engineering, Hebei University of Science and Technology, Shijiazhuang, China; ^2^ Beijing Research Center for Agriculture Standards and Testing, Beijing Academy of Agriculture and Forestry Sciences, Beijing, China

**Keywords:** colorimetric detection, L-histidine, copper, hydrogen peroxide, artificial enzyme

## Abstract

L-Histidine (L-His) is an essential amino acid, which is used to synthesize proteins and enzymes. The concentration of L-His in the body is controlled to regulate tissue growth and repair of tissues. In this study, a rapid and sensitive method was developed for colorimetric L-his detection using Cu^2+^ ions to inhibit the oxidation of 3,3′,5,5′-tetramethylbenzidine (TMB)–H_2_O_2_ system. H_2_O_2_ can oxidize TMB to oxTMB in the presence of copper, and the change in color from colorless (TMB) to blue (oxTMB) is similar to that observed in the presence of peroxidase. However, because the imidazole ring and carboxyl group of L-His can coordinate with Cu^2+^ ions to form stable L-His–Cu^2+^ complexes, the color of the TMB–H_2_O_2_ solution remains unchanged after the addition of L-His. Therefore, because L-His effectively hinders the colorimetric reaction of TMB with H_2_O_2_, this assay can be used to quantitatively determine the concentration of L-His in samples. Under optimized conditions, our colorimetric sensor exhibited two linear ranges of 60 nM to 1 μM and 1 μM to 1 mM for L-His detection and a detection limit of 50 nM (S/N = 3); furthermore, the assay can be performed within 20 min. Moreover, the proposed assay was used to determine the concentration of L-His in urine samples, suggesting that this convenient and label-free colorimetric method presents promising applications in bioanalytical chemistry and clinical diagnosis.

## Introduction

L-Histidine (L-His) levels in the body are typically controlled because L-His regulates the critical physiological functions, such as tissue growth and the transmission of metal elements (Kong et al., 2011). However, inadequate concentrations of L-his in the body can cause chronic kidney disease, primarily by inducing an impaired nutritional state (Huang and Tseng, 2009; Hu et al., 2014). Furthermore, an L-His deficiency can cause Friedreich ataxia, epilepsy, Parkinson’s disease, and abnormal erythropoiesis (Li et al., 2013). Conversely, high concentrations of L-His in physiological fluids (serum and urine) can induce metabolic disorders or histidinemia (Oliveira et al., 2013). Therefore, L-His detection is critical for diseasing diseases. To date, numerous detection methods have been developed for quantifying L-his in biological fluids, including liquid chromatography (Takeuchi et al., 1985), capillary electrophoresis (Meng et al., 2010), electrochemistry (Nai et al., 2013), resonance light scattering (Chen et al., 2006), surface-enhanced Raman scattering (SERS) (Ye et al., 2013), colorimetery (Hyeokseo and SudeokKim, 2013; Wu et al., 2016), and fluorometry (Karasyova et al., 2004). Although these methods present sensitivity and accuracy, they typically require generally complex equipment and professional operation. Moreover, L-His might be derivatizing and labeling using molecular signaling, which is time consuming and labor intensive. Hence, significant efforts are still required for the development of simple, rapid, highly sensitive, and free label method for L-His detection.

Owing to the change in color induced using a simple and rapid operation, colorimetric assays were designed to quantitatively detect biomolecules *via* naked eye observations and ultraviolet-visible (UV-Vis) spectroscopy (Josephy et al., 1982; Karasyova et al., 2004). Considering its chromogenic characteristics, 3,3′,5,5′-tetramethylbenzidine (TMB) has been extensively used as a colorimetric probe (Zhang et al., 2014; Liang et al., 2020). The change in color of TMB in the presence of H_2_O_2_ catalyzed by peroxidase, such as horseradish peroxidase, is a widely used chromogenic reaction (Liu et al., 2020). However, the natural enzymes are expensive and present low stability, which restricted their application in clinical diagnosis. There, artificial enzymes with peroxidase properties have received increasing attention. To date, numerous artificial enzymes, such as Fe_3_O_4_ (Xing et al., 2020), Au nanoparticles (Deng et al., 2016), carbon quantum dots (Chandra et al., 2019), and metal organic frameworks have been extensively investigated (Zheng et al., 2018). The artificial enzymes reported to date exhibited high catalytic efficiency and good stability and were inexpensive. However, most of the reported artificial enzymes presented intrinsic disadvantage such as complex synthesis processes. Recently, Cu^2+^ ions were used to catalyze the oxidation of TMB to oxTMB in the presence of H_2_O_2_ with high efficiency (Zhang et al., 2014). Inexpensive, stable, and readily available Cu^2+^ ions are good candidates as peroxidase mimetics. Analytical systems comprising TMB, Cu^2+^ ions, and H_2_O_2_ were used to detect uric acids (Lu et al., 2017), dopamine (Wang et al., 2017), and glucose (Li et al., 2019) in biological samples. During the analytical process, analytes are catalytically oxidized in the presence of enzymes to generate H_2_O_2_, which induces a color reaction. To the best of our knowledge, the use of a Cu^2+^-triggered colorimetric assay for L-His detection has not been thoroughly investigated to date.

Because of the presence of the N-coordinating ligands of the imidazole ring and –COOH groups, L-his presents a remarkable affinity for Cu^2+^ ions and can strongly chelate with Cu^2+^ ions to form stable L-His–Cu^2+^ complexes (Elbaz et al., 2008; Zhang et al., 2013; Wang et al., 2017; Cai et al., 2020). Upon adding L-His to the assay, the Cu^2+^ ion-catalyzed TMB oxidation to oxTMB in the presence of H_2_O_2_ was inhibited. This resulted in a color change from blue (oxTMB) to colorless (TMB). Based on the mechanism, in this study, a rapid, convenient, and sensitive colorimetric method was designed.

## Experimental

### Materials

All of these reagents were of analytical grade, and all aqueous solutions were prepared with Milli-Q water (>18.2 MΩ⋅cm). 3,3′,5,5′-Tetramethylbenzidine (TMB) and hydrogen peroxide (H_2_O_2_) were purchased from Aladdin Biochemical Technology Co., Ltd. (Shanghai, China). Cu(NO_3_)_2_·3H_2_O was obtained from Tianjin Bodi Chemical Industry Co. Ltd. (Tianjin, China). Na_2_HPO_4_·12H_2_O, NaH_2_PO_4_·2H_2_O, NaCl, and glucose were all purchased from Tianjin Best Chemical Co. Ltd. (Tianjin, China). L-alanine (L-Ala), L-phenylalanine (L-Phe), L-proline (L-Pro), L-histidine (L-His), and urea were all purchased from Beijing Solaibao Technology Co. Ltd. (Beijing, China). The pH of the solution was measured with a PB-10 pH meter (Sartorius, 91 Germany). UV-Vis absorption spectroscopic measurements were carried out on a TU-1900 spectrophotometer (Beijing Pu Analysis General Instrument Co., Ltd.) with an optical path length of 10 mm.

### Coordination-driven chemistry of L-His and Cu^2+^ ions for colorimetric reaction of 3,5′,5,5′tetramethylbenzidine–hydrogen peroxide system

The coordination-driven chemistry of L-His and Cu^2+^ ions was studied for colorimetric reaction. First, the TMB–H_2_O_2_ system was constructed. Two hundred microliters of 420 μM Cu^2+^ions, 40 μl of 160 mM TMB, and 40 μl of 300 mM H_2_O_2_ were added to 2 ml of phosphate buffer saline (PBS) solution (C = 0.1 M, pH = 5.7) in the absence and presence of 200 μl of 80 μM L-His. The mixture was incubated at 45°C for 20 min. Finally, the adsorption spectrum of the mixture was measured on UV-Vis spectrophotometer equipped with 1-cm path length quartz cuvettes.

### Optimization and performance testing of the detecting system

The coordination-driven chemistry of L-His and Cu^2+^ ions was dramatically influenced by pH. We evaluated the effect of pH on the colorimetric reaction of the TMB–H_2_O_2_ system. Different pH of phosphate buffer saline (PBS) solution (C = 0.1 M) were prepared, including 4.0, 5.0, 5.3, 5.7, 6.0, 7.0, 8.0, and 9.0. Then, 40 μl of 160 mM TMB and 40 μl of 300 mM H_2_O_2_ were added to 2 ml of phosphate buffer saline (PBS) solution with various pH.

Then various concentrations of L-His and equal concentration of Cu^2+^ ions were incubated in PBS buffer solution (pH 5.7) at room temperature for 10 min, and the mixture was added into the TMB–H_2_O_2_ system. The final concentration of Cu^2+^ was 30 μM with various concentration of L-His. The mixture was incubated at 45°C for 20 min and then measured by UV-vis.

Meanwhile, the effect of interferents on the detection system was investigated with the above conditions with the substitution of L-His into the interferents. As urine detection probe, L-Ala, L-Phe, L-Pro, glucose, NaCl, and urea, as main constituents of urine, were evaluated as interferents (Sadighi et al., 2006; Dutta et al., 2014; Zheng et al., 2015). Besides, the main molecules were all added into the solution of PBS (c = 0.1 M, pH 7.4) to prepare the simulated urine for recovery testing.

## Result and discussion

### Construction and validation of the coordination-driven chemistry of L-histidine and copper-based biosensing system

**FIGURE 1:**
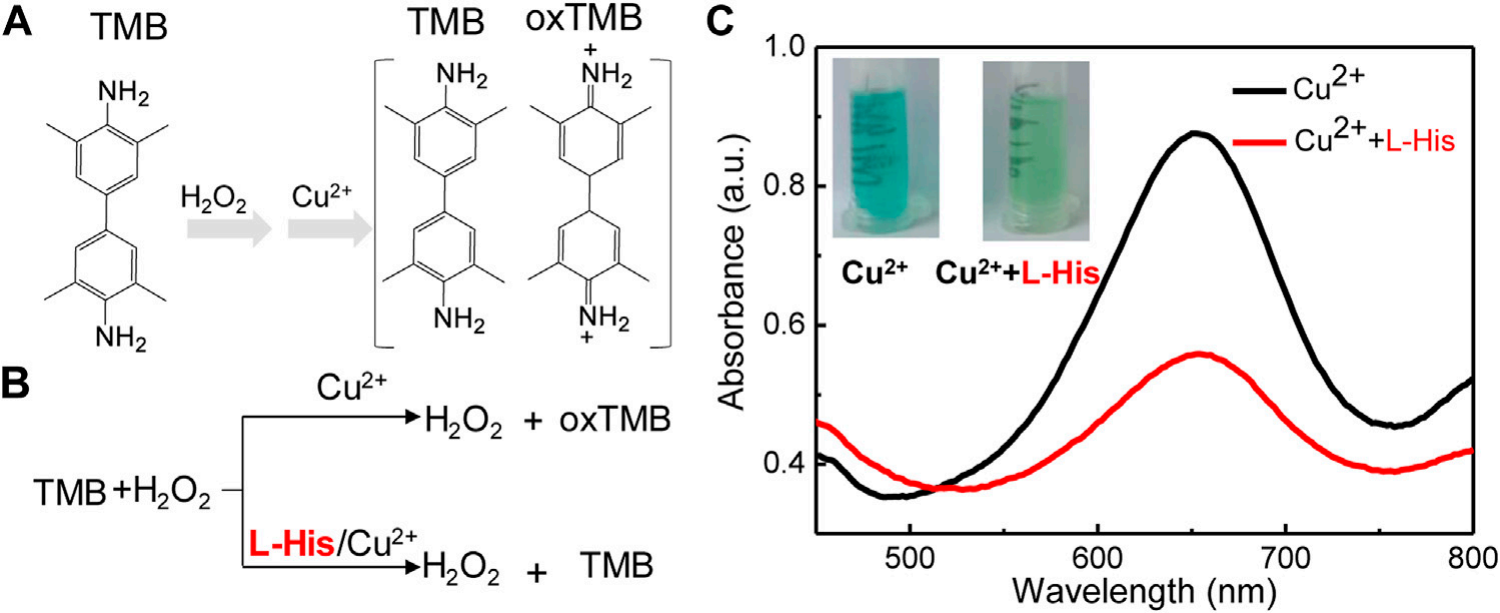
The mechanism and feasibility of the colorimetric platform for L-His detection based on the coordination-driven chemistry of L-histidine (L-His) and Cu^2+^ in the TMB–hydrogen peroxide (H_2_O_2_) system. **(A)** The oxidation of 3,3′5,5′-tetramethylbenzidine (TMB). **(B)** The mechanism of the coordination-driven chemistry of L-His and Cu^2+^. **(C)** Absorption spectra of the TMB + H_2_O_2_ system in the presence of Cu^2+^, Cu^2+^ + L-His, Inset: Photographs corresponding to the curves.

Our biosensing system consisted of two critical reactions: the coordination-driven chemistry of L-His and Cu^2+^ ions and the colorimetric TMB–H_2_O_2_ system (Figure 1). TMB, which served as a colorimetric probe, was oxidized by H_2_O_2_ to oxTMB in the presence of Cu^2+^ ions. During this process, Cu^2+^ ions presented intrinsic catalytic activity for the oxidation of TMB (colorless) to oxTMB (blue) in the presence of H_2_O_2_ (Figure 1A). Upon adding L-His to the biosensing system, the amino groups and hydroxyl groups of L-His chelated with Cu^2+^ ions and formed stable L-His–Cu^2+^ complexes (Zhang et al., 2013). Hence, L-His inhibited the catalytic activity of Cu^2+^ for the oxidation of TMB in the presence of H_2_O_2_, and the color of the TMB–H_2_O_2_ system did not change significantly (Figure 1B). Therefore, the change in color and decrease in absorbance were directly related to the L-His concentration. During the detecting process for the detecting system, the change in color and the decrease in absorbance were both taken as the detection signal of L-His. Hence, we constructed a colorimetric L-His detection sensor based on this mechanism.

To validate the feasibility of the colorimetric sensor for L-His detection, we compared the color and UV-vis spectra of a pristine state of the probe with those of the probe after exposing the TMB–H_2_O_2_ system to L-His. The color of the TMB-H_2_O_2_ was blue, and the highest peak in the UV-vis spectrum of the probe in the presence of Cu^2+^ ions was observed at 652 nm (Figure 1C). The color of the TMB–H_2_O_2_ system changed to light blue in the presence of L-His, indicating that the coordination-driven chemistry of L-His and Cu^2+^ ions affected the oxidation of the TMB–H_2_O_2_ system. Infrared spectrometry (IR) analysis was utilized to confirm the coordination-driven chemistry of L-His and Cu^2+^ ions, and the results indicated that the proposed method was suitable for L-His detection.

### Optimization of experimental conditions

**FIGURE 2:**
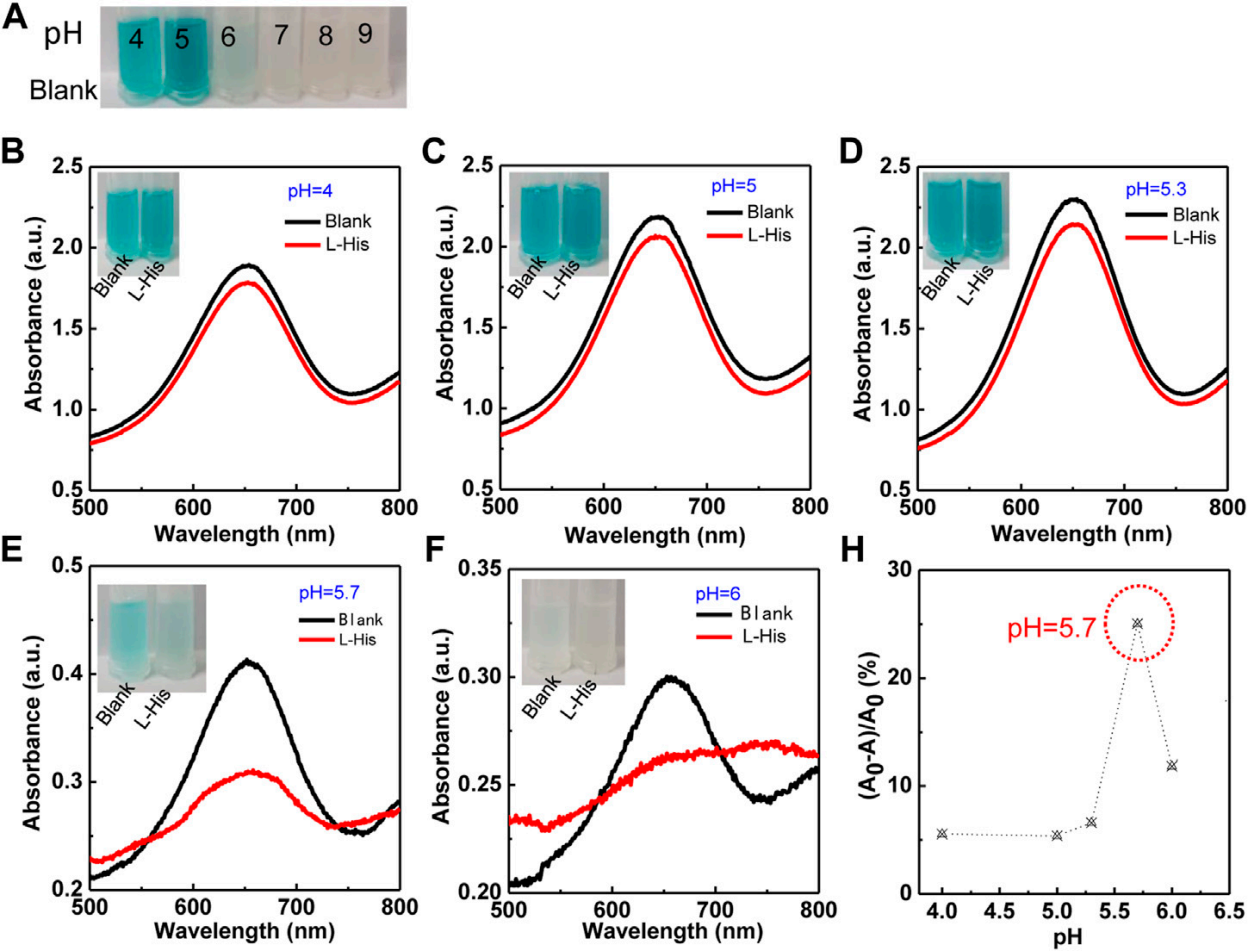
The effect of pH on the colorimetric TMB–H_2_O_2_ system in the presence of Cu^2+^ ions for L-His detection. **(A)** Colorimetric reaction in different pH (the pH were 4, 5, 6, 7, 8, and 9, respectively). **(B–F)** The colorimetric reaction and absorbance intensity change with pH. The pH values were 4, 5, 5.3, 5.7, and 6. The photographs corresponding to the curves. **(G)** The ratio of absorbance ((A_0_-A)/A_0_) was changing with pH.

Because the colorimetric reaction and the coordination of Cu^2+^ ions with L-His were significantly affected by pH, we first evaluated the effects of reaction pH on L-His detection (Figure 2). The colorimetric reaction of the TMB–H_2_O_2_ system was significantly affected by pH (Figure 2A). The color of the TMB–H_2_O_2_ system was blue at pH 4, 5, and 6 and changed to yellow and colorless with the increasing pH to 7, 8, and 9. This indicated that the optimum pH for colorimetric reaction ranged between 4 and 6. Therefore, we subsequently evaluated the effect of pH in the range of 4–6 on the detection assay (Figures 2B–G). The color-changing rate on the TMB–H_2_O_2_ system increased rapidly with increasing pH, reached a plateau at pH >5.7, and then it decreased. The UV-vis absorbances of the highest peak (*λ*_655_) in the absence and presence of L-His were defined as A_0_ and A, respectively. The UV-vis spectra indicated that the absorbance-changing rate of the TMB–H_2_O_2_ system [(A_0_-A)/A_0_] was the highest at pH is 5.7 (Figure 2H). Therefore, pH 5.7 was used as the optimum pH value of the assay buffer of the sensing system.

### Colorimetric assay for L-histidine detection

**FIGURE 3:**
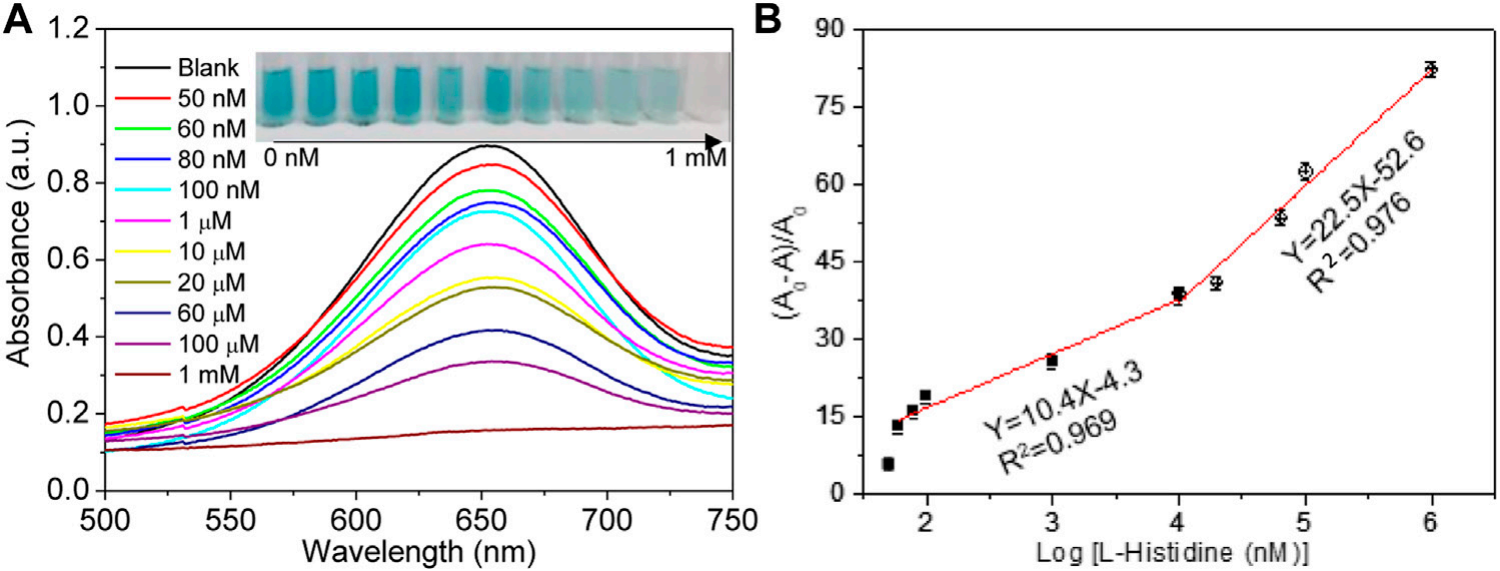
Limit of detection. **(A)** Absorption spectra change in the sensing system with the increasing concentrations of L-his (the concentrations were 0 nM, 50 nM, 60 nM, 80 nM, 100 nM, 1 μM, 10 μM, 20 μM, 60 μM, 100 μM, and 1 mM, respectively). The photographs corresponding to the curves. **(B)** The rate of absorbance changing (A_0_-A/A_0_) at 655 nm change, with the increase in L-His.

Owing to the catalytic effect of Cu^2+^ ions on the TMB–H_2_O_2_ system and the chelating interaction of Cu^2+^ ions and L-His, an assay for rapid and simple L-His detection was fabricated. The change in absorbance at 652 nm of the TMB–H_2_O_2_ system with increasing L-His concentration was analyzed under the optimized experimental conditions (Figure 3A). The absorbance of sensing system decreased with the addition of L-His. Moreover, the photographs of the corresponding solution color were inserted into Figure 3A. Upon increasing L-His concentration, the color of the TMB–H_2_O_2_ system changed from deep blue to colorless. Furthermore, the containment level of 10 μM L-His can be clearly distinguished with the naked eye. In addition (A_0_-A)/A_0_ depended on the concentration of L-His and increased linearly upon increasing L-His concentration in the range of 60 nM to 1 μM and 1 μM to 1 mM, respectively (inset in Figure 3B). The limit of detection of the sensing system was 50 nM (S/N = 3), and the linear equations describing the dependence of (A_0_-A)/A_0_ on the L-His concentration in the aforementioned L-His concentration ranges were y = 22.5 × −52.6 and y = 10.4 × −4.3, respectively (regression coefficient of 0.976 and 0.969, respectively).

To effectively exhibit the proposed method, the method was listed to compare with other methods (colorimetric and fluorescent platform based on nanomaterials) in Table 1. Compared with other colorimetric or fluorescent assay, the colorimetric strategy in this study has lower detection and wider analytical range.

**TABLE 1:** Comparison of different methods for L-histidine (L-His) detection.

Analytical method	Linear range	LOD	References
Colorimetric probe of TMB-H_2_O_2_	60 nM–1 μM	60 nM	This work
	1 μM–1 mM		
Colorimetric probe of DNAzyme cascade	5 μM–100 mM	50 μM	Pan et al. (2018)
Fluorescent probe of azide and alkyne cycloaddition (CuAAC) reaction	0.5–100 μM	76 nM	Qiu et al. (2013)
Fluorescence probes of dopamine functionalized–CdTe quantum dots	1.0–100 μM	500 nM	Shi et al. (2014)
Fluorescent probe of nitrogen-doped carbon nanoparticles	0.5–60 μM	150 nM	Zhu et al. (2016)
Colorimetric probe of G-quadruplex-Cu metalloenzyme	10 nM–1.0 μM	10 nM	Wu et al. (2016)

### Selectivity of the colorimetric detection and artificial urine

**FIGURE 4:**
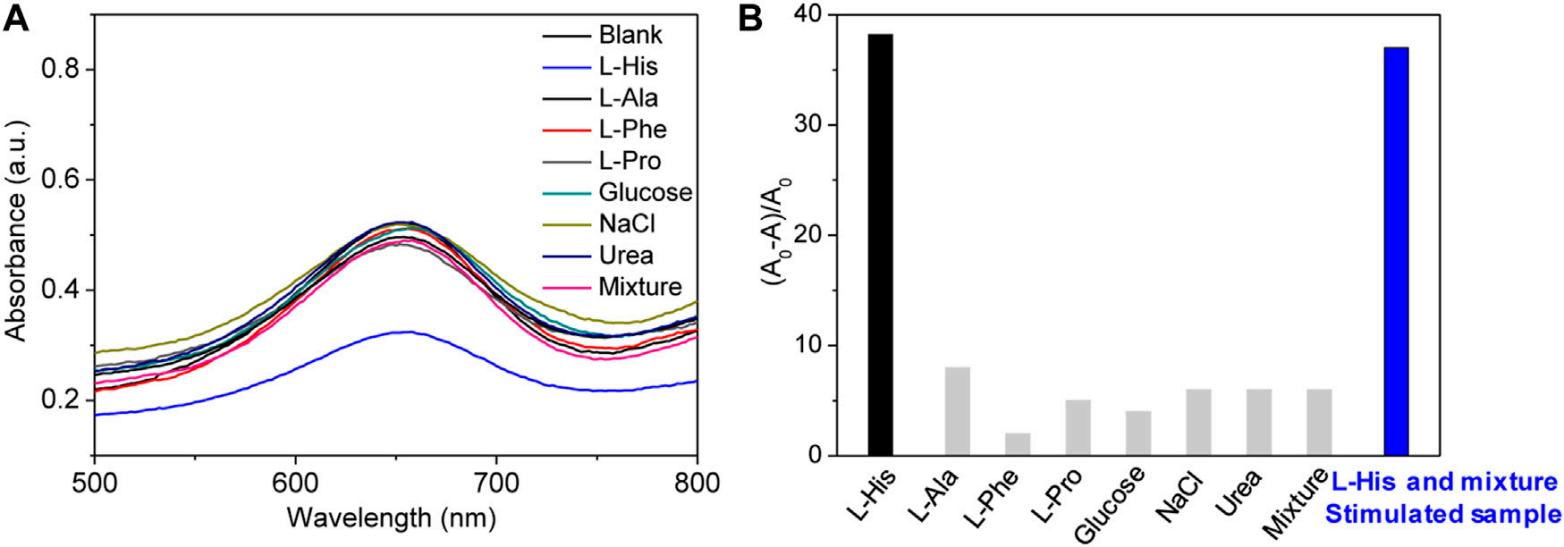
Selectivity and simulated samples detection. **(A)** Absorption spectra of the sensing system in the presence of different molecules, including L-His, L-alanine (L-Ala), L-proline (L-Pro), L-phenylalanine (L-Phe), glucose, NaCl, and urea (the concentrations of the molecules were all 80 μM). **(B)** The rate of absorbance changing (A_0_-A/A_0_) at 655 nm change with different molecules.

The selectivity of the proposed colorimetric sensing system should be evaluated for real or simulated samples because real samples are complex. In particular, amino acids present in real samples can interfere with the quantitative analysis of L-His because their properties and structure are similar to those of L-His. Therefore, we selected L-alanine, L-proline, and L-phenylalanine as interferents to be evaluated because they are the primary components of urine. Meanwhile, the effect of glucose, urea, and NaCl were also studied because they are also the main component in urine (Figure 4). The change of absorbance and (A_0_-A)/A_0_ were presented in Figures 4A,B, respectively. Because the tested amino acids and biomolecules did not present significant signals, we concluded that our platform presented good selectivity for L-His, which is the most distinct difference in the structure between L-His and other amino acids. Other amino acids cannot chelate interaction with Cu^2+^ ions and have no effect on the colorimetric reaction of the TMB–H_2_O_2_ system.

### Detection of L-histidine in simulated samples

To illustrate the practical application of colorimetric sensor for L-His detection, the simulated urine was prepared according to the previous literature (Sadighi et al., 2006; Dutta et al., 2014; Zheng et al., 2015). Briefly, L-Ala, L-Phe, L-Pro, glucose, NaCl, and urea were all added into the solution of PBS (c = 0.1 M, pH 7.4). Besides, different L-His concentrations were also added into the simulated urine. We used the standard addition method to obtain the L-His concentrations of simulated samples (Table 2). The recovery values were 102.0%, 95.3%, and 98.5%, respectively. Meanwhile, the relative standard deviations (RSDs) were 3.8, 4.2, and 2.1. The recovery values were between 95.3% and 102.0%, and the RSDs were no more than 4.2%. These results demonstrated that the proposed colorimetric sensor had a promising application for glyphosate detection in real samples.

**TABLE 2:** The testing result of different L-histidine concentrations in simulated human urine (*N* = 3).

Sample no	Added (μM)	Found (μM)	Recovery (%)	R.S.D. (%, *n* = 3)
1	60	61.2	102.0	3.8
2	80	76.2	95.3	4.2
3	100	98.5	98.5	2.1

## Conclusions

In conclusion, the proposed platform as a colorimetric sensor can effectively detect L-His with simple and rapid operation. Owing to the coordination-driven chemistry and the strong chelating interaction between Cu^2+^ ions and L-His, the colorimetric sensor presents highly sensitive and selective detection of L-His with a detection limit of 50 μM by the naked eye. Meanwhile, the method did not need any label and is easy to obtain, and can be finished within 20 min. Compared with other TMB–H_2_O_2_ systems, the sensing platform is simple without enzymes and the complicated operation for biomolecules. In addition, we found that the interferents of urine have no effect on colorimetric platform. The detecting system was successfully used to detect L-His in the simulated samples and exhibited a great promise for practical application in biological and clinical diagnosis fields.

## Data Availability

The original contributions presented in the study are included in the article/Supplementary Material, further inquiries can be directed to the corresponding authors.
